# HLA-DRB1 as a risk factor in children with autoimmune hepatitis and its relation to hepatitis A infection

**DOI:** 10.1186/1824-7288-36-73

**Published:** 2010-11-10

**Authors:** Amel AM Elfaramawy, Reham M Elhossiny, Amal A Abbas, Heba M Abdel Aziz

**Affiliations:** 1 The pediatrics department, Faculty of medicine, Ain Shams University Cairo, Egypt; 2 The clinical pathology department, Faculty of medicine, Ain Shams University, Cairo, Egypt

## Abstract

**Background:**

The human leukocyte antigens (HLAs) are proteins found in the membranes of nearly all nucleated cells. People with certain HLA antigens are more likely to develop certain autoimmune diseases. The aim of this study was to determine the frequency of HLA-DRB1 in children with autoimmune hepatitis (AIH) as a risk factor for occurrence, its relation to preceding hepatitis A infection and treatment outcome.

**Subjects and methods:**

25 children with AIH were subjected to HLA-DRB 1 typing performed by sequence specific oligonucleotide probe technique and compared to HLA-DRB1 found in 548 normal populations.

**Results:**

The most frequent alleles found in our children with AIH were HLA-DRB1*13 (36%), HLA-DRB1*04 (18%) and HLA-DRB1*03 (14%). HLA-DRB1*13 was significantly more frequent in AIH patients compared to controls. In type I AIH patients HLA-DRB1*13 was the most frequent allele (32.4%), followed by HLA-DRB1*04 in (20.6%) and HLA-DRB1*03 in (14.7%), While in type II, the most frequent alleles were HLA-DRB1*13 in (40%), HLA-DRB1*07 (20%) and HLA-DRB1*15 in (20%). HLA-DRB1*12 was significantly more frequent in AIH patients with positive Hepatitis A IgM than in patients with negative hepatitis A IgM. No statistically significant difference between partial responders and complete responders to treatment as regards HLA-DRB1 subtypes.

**Conclusion:**

It is concluded from the previous study that HLA-DRB1*13 may be a susceptibility allele for the occurrence of autoimmune hepatitis in our population. HLA-DRB1*07 and HLA-DRB1*15 may be susceptibility alleles for occurrence of autoimmune hepatitis type 2. HLA-DRB1*12 association with AIH in patients triggered by hepatitis A needs further studies.

## Introduction

Auto-immune hepatitis (AIH) is a chronic liver disorder of unknown cause that leads to cirrhosis and liver failure when untreated. The disease usually affects females and is characterized by the presence of circulating auto antibodies and by severe interface hepatitis on liver biopsy [1].

Two types of AIH have been proposed on the basis of serologic markers, type 1-AIH is the most common form worldwide while type 2-AIH occurs mainly in children and in Europe [2].

Although the etiology of AIH is unknown, both genetic and environmental factors are involved in its expression [3]. Strong evidence suggests that defects in immunologic control of auto reactivity play a role in AIH pathogenesis [4].

HLA antigens are the major determinants used by the body's immune system for recognition and differentiation of self from non-self. There are many different major histocompatibility (HLA) proteins, and each person possesses only a small, relatively unique set that is inherited from their parents [5].

Autoimmune diseases are the result of interplay between predisposing genes and triggering environmental factors, leading to loss of self-tolerance and an immune-mediated destruction of autologous cells and/or tissues. Genes in the HLA complex are among the strongest predisposing genetic factors [6]. In auto-immune hepatitis patients, HLA DRB positive patients are more likely to have the disease than other patients [7]. Although, drugs and toxins may theoretically affect immune homeostasis and trigger AIH, primarily viruses have been studied in this context [8].

The aim of this study was to determine the frequency of HLA-DRB1 in patients with autoimmune hepatitis as a risk factor for occurrence, its relation to preceded HAV infection, and treatment outcome.

## Patients and Methods

This study was conducted on 25 children with AIH recruited from patients regularly attending the Hepatology Clinic, Children's Hospital; Ain Shams Faculty of Medicine and Prof. Yassin Abd El-Ghaffar Charity Centre for Liver Diseases and Research, both are tertiary referral centers. It was performed according to the recommendations of the Ethics Committee of Ain Shams University Hospitals. An informed consent was obtained from the children's guardians.

The diagnosis of AIH was based on the International Scoring Criteria for auto-immune hepatitis [9] evaluated before the start of any immunosuppressant treatment. Patients with primary sclerosing cholangitis or overlap syndrome were excluded from the study.

All patients included were subjected to proper history taking, thorough clinical examination

- Laboratory investigations were done for all patients and included complete blood count, serum ALT, AST, total bilirubin, direct bilirubin, prothrombin time, serum albumin and serum protein electrophoresis.

-Abdominal ultrasound was done for all patients and liver biopsy was done for initial evaluation in 19 patients and after the start of treatment and stabilization of the bleeding profile in 6 patients.

- HLA-DRB typing by sequence specific oligonucleotide probe (SSOP) technique in which 5 ml blood on sterile EDTA tubes was collected from each patient. DNA extraction was done using the DNA isolation kit (minikit, Qiagen, Hilden Germany). HLA-DRB typing was done using Dynal RELITM SSO HLA-DRB typing kit (Dynal Biotech Ltd., UK).

The principal is based on three major processes:

1) PCR amplification reactions which were done using the thermal cycler to produce an amplified biotinylated DNA sequence termed an amplicon utilizing biotinylated primers. PCR was done applying the following protocol: denaturation at 95 degree for 15 seconds, primer annealing at 60 degree for 45 seconds and extension at 72 degree for 15 seconds. This process was repeated for 35 cycles. 2) Hybridization step in which the amplicons were chemicallly denatured to form single stranded DNA, these were added to nylon membrane that contains an array of immobilized sequence-specific oligoneuclotide (SSO) probes. The biotin-labeled amplicons then bind (hybridise) to those SSO probes that contain a complementary target sequence and thus "captured" on to membrane strip. 3) Detection of the amplicon-probe complex was done using a colorimetric reaction. Interpretation of the results was done using the Dynal RELITM SSO pattern matching program [10].

HLA typing of our patients was compared to a published study done by **Khalil et al **[11] which was performed in Ain Shams University Specialized Hospital, where 548 Egyptians were investigated for the most frequent HLA-DR alleles among the Egyptian population using sequence specific oligonucleotide probe (SSOP) technique. It is considered a weakness point to use controls from different study, yet the included subjects represent the normal population in our country as this hospital is a referral center.

- Response to treatment was evaluated as follows "Complete remission" is indicated by the absence of symptoms, and return of serum ALT or AST, bilirubin and immunoglobulin values to normal within 1 year, or histologic improvement to normal or minimal inflammatory activity on liver biopsy. "Treatment failure" is defined as deterioration in a patient's clinical condition, laboratory tests, or histologic features despite intensive therapy." Incomplete response to treatment" is defined as an improvement that is insufficient to satisfy remission criteria [9].

### Statistical methods

SPSS statistical software package was used for data analysis. Date was expressed as Mean ± SD for quantitative measures and both number and percentage for categorized data. Comparison between two independent means for parametric data was done using student test. Comparison between two independent groups for non-parametric data was done using Wilcoxon Rank Sum test. Wilcoxon signed rank test for comparison between two dependent groups for non-parametric data. Chi-square test used to study the association between 2 variables, or comparison between 2 independent groups as regards the categorized data. The probability of error at 0.05 was considered significant.

## Results

The included children with AIH were 17 females (68%) and 8 males (32%), their ages ranged from 6 to 18 years with a mean of 13.2 ± 3.8 years. The mean duration of illness was 4.4 ± 3.1 years.

The clinical, biochemical, and histological features at presentation are reported in (Table 1). All our patients were symptomatic at presentation and the most frequent type of onset was acute hepatitis with cholestatic jaundice and elevated liver enzymes (64%) while chronic presentation with cirrhotic liver and portal hypertension was found in 36%. Type 1 AIH was predominant in children as detected by positive ANA and/or ASMA in 68%, while type 2 was characterized by the presence of anti- LKM in only 20% and 12% were seronegative, according to the International Scoring Criteria for auto-immune hepatitis, the diagnosis was definite in 88% and probable in 12%. Concomitant autoimmune thyroiditis was detected in 3 (15%) children out of 20, who were subjected to thyroid auto antibodies testing.

**Table 1:** Clinical, biochemical, and histological features of autoimmune hepatitis at presentation

Sex (F/M)	17/8
Type 1/type 2/seronegative AIH	17/5/3
Presentation:	
Acute	16 (64%)
Chronic	9 (36%)
ALT(IU/L) × normal	10 × n
AST(IU/L) × normal	9 × n
Alb(g/dL)	3.5 ± 0.8
Gammaglobulin (g/dl)	3.3 ± 1.8
Total Bilirubin (mg/dl)	4.9 ± 2.8
Direct bilirubin (mg/dl)	3.1 ±1.95
ANA	9(36%)
ASMA	17 (68% )
Anti-LKM	5(20%)
Autoimmune thyroiditis ( 20)	3(15%)
Hepatitis A IgM +ve	6 (24%)
Liver biopsy:	
Interface hepatitis	23(92% )
Fibrosis	18 (72%)
Cirrhosis	7(32%)
Complete response	16(64%)
Partial response	7(28%)
Treatment failure	0
Non compliance	2(8%)

Nearly quarter of our patients were presented at the beginning with a picture of acute hepatitis that was diagnosed as hepatitis A evident by positive hepatitis A IgM. Based on the clinical course as those patients had unusual prolonged (more than one month) or protracted course of hepatitis A, the diagnosis turned to be AIH according to the International Autoimmune Hepatitis Group's system. They all had type 1 AIH. The rest of the patients were either vaccinated or negative for HAV IgM.

Liver biopsy showed that 92% of them had severe inflammation, while fibrosis was present 72% and cirrhosis was evident at presentation in 32%.

Complete response was achieved in 64% of the included children while 28% only partial response could be achieved and no one showed failure to response however 8% were non compliant to treatment.

The most frequently observed class II alleles in the group of patients were HLA-DRB1*13 as it constituted 36% of alleles, then HLA-DRB1*04( 18%), HLA-DRB1*03 ( 14%). The most frequent alleles found in the control group were HLA-DRB1*03 ( 19%) then HLA-DRB1*13 and HLA-DRB1*04 as each constituted 16% of alleles. Only HLA-DRB1*13 was significantly prevalent in our patients with AIH (Table 2).

**Table 2:** Comparison between the studied cases and control group as regards HLA-DRB1*

Alleles	No of alleles in cases = 50		No of alleles in controls = 1096		P
	
HLA-DRB1*	No	%	No	%	
01	3	6	55	5	>0.05

03	7	14	214	19	>0.05

04	9	18	175	16	>0.05

07	3	6	66	6	>0.05

08	2	4	22	2	>0.05

11	4	8	132	12	>0.05

12	1	2	22	2	>0.05

13	18	36	175	16	<0.001

15	3	6	77	7	>0.05

Although HLA-DRB1*13 was the most frequent allele in type 2 AIH, yet statistically HLA-DRB1*07, HLA-DRB1*15 alleles were significantly more frequent in type 2 AIH versus type 1 (table 3). The group of children in which the diagnosis of AIH was probable according to the scoring system showed that their HLA-DRB1 allels were 3/13 in one patient, 4/13 in the second one and 1/13 in the third one which were not different from patients with confirmed diagnosis.

**Table 3:** Comparison between Types of AIH as regards HLA-DRB1*

Alleles	Type 1 (no = 34)		Type 2 (no = 10)		Sero -ve (no = 6)		Type 1 vs. Type 2	Type 1 vs. sero -ve	Type 2 vs. sero -ve
HLA-DRB1*	**No**	**%**	**No**	**%**	**No**	**%**	**P**	**P**	**P**

1	2	5.9	0	0	1	16.7	>0.05	>0.05	>0.05

3	5	14.7	1	10	1	16.7	>0.05	>0.05	>0.05

4	7	20.6	1	10	1	16.7	>0.05	>0.05	>0.05

7	1	2.9	2	20	0	0	<0.05	>0.05	>0.05

8	2	5.9	0	0	0	0	>0.05	>0.05	>0.05

11	4	11.8	0	0	0	0	>0.05	>0.05	>0.05

12	1	2.9	0	0	0	0	>0.05	>0.05	>0.05

13	11	32.4	4	40	3	50	>0.05	>0.05	>0.05

15	1	2.9	2	20	0	0	<0.05	>0.05	>0.05

HLA-DRB1*13 was the most prevalent allele in autoimmune hepatitis patients with positive HAV IgM in this study however HLA-DRB1*12 was the only statistically significant allele in those patients compared to patients with negative hepatitis A IgM and no significant difference as regards other alleles was found (Table 4).

**Table 4:** Comparison between patients with Hepatitis A (+ve IgM) and patients with Hepatitis A (-ve IgM) as regards HLA-DRB1*

Alleles	Hepatitis A +ve IgM No = 12		Hepatitis A -ve IgM No = 38		P
	
HLA-DRB1*	No	%	No	%	
01	1	8.3	2	5.3	>0.05

03	0	0	7	18.4	>0.05

04	1	8.3	8	21	>0.05

07	1	8.3	2	5.3	>0.05

08	1	8.3	1	2.6	>0.05

11	2	16.7	2	5.3	>0.05

12	1	8.3	0	0	<0.05

13	4	33.3	14	36.8	>0.05

15	1	8.3	2	5.3	>0.05

Although HLA-DRB1*03 was frequently found in partial responders to treatment and HLA-DRB1*04 was frequently found in complete responders, yet this was not of statistical significance neither do other HLA-DRB1* subtypes.

## Discussion

Autoimmune hepatitis is a chronic hepatitis of unknown etiology characterized by immunologic and auto immunologic features [12]. High-resolution DNA-based techniques have indicated that the principal susceptibility factors for autoimmune hepatitis associated with ASMA and ANA expression reside on the *DRB1 *gene [13].

There is substantial evidence favoring hepatitis A virus (HAV) as an etiological factor in AIH. This includes individual case reports of persistent hepatic inflammation, with serological and histological features of AIH following proven infection with HAV, and the development of autoantibodies to hepatic constituents during and after HAV infection [14].

The aim of this study was to determine the frequency of HLA-DRB1 in patients with autoimmune hepatitis as a risk factor for occurrence, its relation to preceded HAV infection, and treatment outcome.

Type 1 AIH is the most common form worldwide, while Type 2 AIH occurs mainly in children and in Europe [15]. However in our study although performed on children, 68% were categorized as type 1 AIH and 20% as type 2. This disagree with **Muratori et al **[16], who published the Bologna experience of autoimmune hepatitis in Italy and reported that among 28 included children 24 had AIH type 2 but this can be explained by different ethnic background.

In 64% of our patients the presentation of the disease was acute marked by signs and symptoms of acute hepatitis, whereas in the remaining 36% the patients presented with overt chronic liver disease with portal hypertension.

The modes of presentation differ largely in different studies as **Mieli-Vergeni and Vergani **[17] reported that the course of the disease may be fluctuating with flares and spontaneous remission, a pattern that may result in delayed referral and diagnosis. **Kogan et al **[18] found that 34% of patients were asymptomatic at presentation and typically, discovered during routine general medical examination that includes the screening of liver tests and **Muratori et al **[16] in their study found that 42.8% of children patients were presented with acute hepatitis while 14% were asymptomatic and 7.2% of their patients had insidious onset. The higher percentage of symptomatic patients in our study can be explained by the fact that all the included patients were recruited from referral centers so they are likely to represent those with more severe disease.

Cirrhosis was present in the liver biopsy in 32% and fibrosis in 72% of the patients who were biopsied for histopathological diagnosis. **Ferrari et al **[19] stated that the presence of overt cirrhosis in one quarter of patients at the time of diagnosis suggests that the disease has a subclinical expression lasting several months or even years.

Nearly quarter of our patients were presented at the beginning with acute hepatitis A as evident by positive hepatitis A IgM and the diagnosis turned to be AIH in those patients based on the clinical course and according to the International Autoimmune Hepatitis Group's system. HLA-DRB1*12 was statistically significant in those patients than patients with negative hepatitis A IgM and no significant difference as regards other alleles was found, but due to small number, this needs further confirmation.

Attention has focused on the theory of molecular mimicry between the infectious particle and the liver constituent against which the resulting antibodies react. Such mimicry between a viral and a hepatic epitope has been demonstrated [20]. In relatives of patients with AIH, **Vento *et *al **[21] demonstrated an intrinsic defect in suppressor-inducer T-cells mediating immune reactivity to a liver antigen (asialoglycoprotein receptor-ASPGR), and described the development of AIH following sub-clinical exposure (seroconversion to HAV) with a simultaneous rise in anti-ASGPR antibodies. **Urganci et al **[22] report a 6 year old boy with autoimmune hepatitis and autoimmune hemolytic anemia following HAV infection and **Tabak et al **[23] reported a 21-year-old female with acute HAV that turned to be AIH and concluded that, prolonged HAV infection and AIH may not only trigger each other but also deteriorate the liver histology.

This raises the question of the ability of hepatitis A vaccine to protect genetically susceptible patients (other family members) from developing autoimmune hepatitis however; **Berry and Smith-Laing **[24] reported a case of hepatitis A vaccine associated with autoimmune hepatitis and concluded that the recrudescence of this case following vaccination with inactivated HAV adds to the evidence that HAV may trigger autoimmunity against the liver and should not be interpreted as a side effect based on the vaccine's track record of safety, but reveals potential connections between two apparently disparate liver pathologies.

HLA-DRB1*13 was a susceptibility allele for the occurrence of AIH in our population, in accordance **Bittencourt et al **[25] as they found that genetic susceptibility to AIH linked to HLA-DRB1*13 but, HLA-DRB1*03 allele was also found in the Brazilian population and indicated that different HLA antigens confer the susceptibility of AIH types I and II. In Caucasian patients, those with HLADR3 and DR4 are independently susceptible to AIH while, DR4 is predominant in Japanese patients and there are no Japanese patients with DR3 [26].

**Fainboim and associates **[27] stated that HLA DRB1*13 is a risk factor for type 1 AIH in South America, and HLA DRB1*03 and DRB1*04 are independent risk factors for the disease in white North Americans. Moreover they stated that HLADRB1*13 has been implicated in the clearance of viral infection and in the immune response against viral proteins. Its presence may select individuals for protracted exposure to viral and hepatic antigens that favor development of the disease. The predominant association of HLA DRB1*13 with type 1 autoimmune hepatitis in Brazil and Argentina suggests that HLA DRB1*13 favors the presentation of triggering antigens that are common to this region, and the hepatitis A virus may be one of several such agents, may be this can explain the prevalence of type 1 AIH among our children and not type 2 as we are also endemic area for Hepatitis A.

There was no statistically significant difference between partial responders and complete responders to treatment of AIH according to HLA-DRB1*. However **Montano-Loza and associates **[28] found that the deterioration during conventional corticosteroids therapy occurs infrequently in patients with definite type I auto-immune hepatitis at presentation. Treatment failure is associated with onset at an early age, acute presentation, hyperbilirubinemia and HLA-DRB1*03. **Muratori et al **[16] found that type I and type II AIH are one and the same disease, has similar features in male and female patients, HLA-DRB1*04 positive patients are more likely to achieve complete remission. Continuous on low dose steroids are necessary to maintain remission, significantly reducing the risk of disease progression.

It was concluded from this study that HLA-DRB1*13 may be a susceptibility allele for the occurrence of autoimmune hepatitis in our population. HLA-DRB1*07 and HLA-DRB1*15 may be susceptibility alleles for occurrence of autoimmune hepatitis type 2. HLA-DRB1*12 association with AIH in patients triggered by hepatitis A needs further studies.

## Competing interests

The authors declare that they did not receive any source of support, including pharmaceutical and industry support or any funding from any organization.

## Authors' contributions

AE: conceived, designed the study, collection of demographical and clinical data of the children, analysis and interpretation of data, drafting and revising the manuscript, R E: acqusition of data, helped in collection of demographical and clinical data of the children and participated in revising the manuscript, A A: carried out the laboratory studies, H A: helped in collection of demographical and clinical data of the children participated in drafting. All authors read and approved the final manuscript.

## Authors' information

AE: Assistant Professor of Pediatrics and Pediatric hepatology, Ain Shams University, Cairo, Egypt; R E:Lecturrer of Pediatrics, Ain Shams University, Cairo, Egypt; A A: Assistant Professor of Clinical Pathology, Ain Shams University, Cairo, Egypt.; H A: Master degree in pediatrics.
